# Validation of a CE-IVD, urine exosomal RNA expression assay for risk assessment of prostate cancer prior to biopsy

**DOI:** 10.1038/s41598-022-08608-z

**Published:** 2022-03-21

**Authors:** Alexander Kretschmer, Holger Kajau, Eric Margolis, Ronald Tutrone, Tobias Grimm, Matthias Trottmann, Christian Stief, Georg Stoll, Christian A. Fischer, Claudia Flinspach, Anja Albrecht, Lisa Meyer, Tina Priewasser, Daniel Enderle, Romy Müller, Phillipp Torkler, Jason Alter, Johan Skog, Mikkel Noerholm

**Affiliations:** 1 Ludwig-Maximilians University Munich, Munich, Germany; 2 SRH Wald-Klinikum Gera GmbH, Gera, Germany; 3 Department of Urology, Hackensack Meridian School of Medicine, Nutley, USA; 4 Chesapeake Urology Associates, Baltimore, MD USA; 5 Urologische Gemeinschaftspraxis Kaufbeuren, Kaufbeuren, Germany; 6 Urologie und Andrologie am Promenadeplatz, Munich, Germany; 7 Exosome Diagnostics, Waltham, MA USA

**Keywords:** Molecular biology, Predictive markers, Prostate cancer

## Abstract

Improved risk stratification of patients suspected of prostate cancer prior to biopsy continues to be an unmet clinical need. ExoDx Prostate *(IntelliScore)* “EPI” is a non-invasive urine test utilizing RNA from exosomes to provide a risk score that correlates with the likelihood of finding high grade prostate cancer at biopsy. Here, we present the results from a prospective clinical validation study of EPI-CE, a CE-marked *in-vitro* diagnostic (IVD) assay, specifically developed for use in European clinical laboratories. The study (NCT04720599) enrolled patients with ≥ 50 years, PSA 2–10 ng/mL, prior to MRI, who were scheduled for initial biopsy. First catch urine samples were collected from participants without prior digital rectal examination or prostate massage. Exosomal RNA was isolated and expression levels of three biomarkers ERG, PCA3 and SPDEF were analyzed according to the EPI-CE Instructions For Use. In the study cohort of N = 109 patients, EPI-CE was validated to have a Negative Predictive Value of 89%, a Sensitivity of 92% and a superior performance to two commonly used multiparametric risk calculators (PCPT and ERSPC) in both Receiver Operating Characteristics with a higher Area Under the Curve for EPI-CE 0.67 (95% CI 0.56–0.77) versus PCPT 0.59 (95% CI 0.47–0.71) and ERSPC 0.60 (95% CI 0.49–0.72) and higher Net Benefits analysis across a wide range of risk acceptance levels. This is the first clinical study reporting on the performance of EPI-CE. We demonstrate that EPI-CE provides information beyond standard clinical parameters and provides a better risk assessment prior to MRI, of patients suspected of prostate cancer, than the commonly used multiparametric risk calculators.

## Introduction

The EAU recently published their recommendations on early detection of prostate cancer, arguing that addition of risk stratification tools has resulted in a more favorable balance between the harms and benefits of PSA screening^1^. According to these recommendations, multivariable risk prediction models should be deployed as early risk stratification tools followed by magnetic resonance imaging (MRI) for higher risk patients to determine who should proceed with prostate biopsy.

We previously published on the development of a urine exosome gene expression test, ExoDx Prostate *(Intelli-Score)* (EPI-LDT, Exosome Diagnostics, Waltham, MA 02451, USA), which consistently outperforms the multivariable risk prediction models in identifying high-grade PCa (HGPCa) of Grade Group (GG) 2 or greater in studies in the US^2–5^. The EPI test relies on exosomes, which are small lipid bilayer vesicles secreted from living cells. Exosomes contain RNA, DNA and protein from their cell of origin and can be detected in a variety of biofluids such as blood, cerebrospinal fluid, and urine. Exosomes are particularly useful for RNA expression analysis because they protect RNA in the biofluid and during sample processing^6–9^. The EPI test utilizes exosome RNA expression levels of the three genes ERG, PCA3 and SPDEF and is a standalone test that does not incorporate any other parameters or clinical features to assess the risk of HGPCa. Further, the EPI test is a non-invasive urine test that does not require digital rectal examination (DRE) or prostate massage prior to urine sample collection, and which is intended for men, 50 years or older, presenting for initial biopsy with a PSA level of 2–10 ng/mL. Most recently, the EPI score was shown to be associated with risk of adverse pathology on radical prostatectomy in men with a Gleason Grade Group 1 positive biopsy, suggesting that the EPI test might also find utility in additional clinical indications^10^. The EPI test has been available in the United States as a laboratory developed test for several years, following the regulations of the Clinical Laboratory Improvement Act (CLIA), but differences in infrastructure, languages and local national health care systems in European countries necessitates a more localized laboratory testing approach.

In this study we report on “EPI-CE”, a newly developed *in-vitro* diagnostic (IVD) version of the EPI test, which follows the European requirements for CE-marking of IVDs^11^, making it available to clinical laboratories, doctors and patients across Europe for the first time. The biomarkers and working principle of the EPI-CE test is identical to the EPI test offered in the US. To date, there are no publications investigating the clinical performance of EPI-CE. To address this paucity of data, here we report on the performance of EPI-CE in a prospective clinical validation study.

## Materials and methods

### Clinical study design

The clinical study was performed as a prospective, single arm study, enrolling subjects scheduled for prostate biopsy due to suspicion of prostate cancer, who were ≥50 years, with PSA levels of 2–10 ng/mL, from sites in Germany, United Kingdom and United States (NCT04720599). To ensure independent estimates of EPI-CE performance prior to MRI, subjects were required to be MRI-naïve at the time of biopsy. Patients were excluded if they had symptoms of urinary tract infection (including prostatitis), a history of prostate cancer, or a history of invasive transurethral treatments within six months prior to enrollment. The outcome of EPI-CE urine analysis was compared to the corresponding tissue biopsy histopathology, as the reference test for diagnosis of prostate cancer. The performance of the EPI-CE test was compared to that of two commonly used multiparametric risk calculators (PCPT and ERSPC) as the gold standards for risk stratification of patients prior to biopsy. No randomization was performed. The primary objectives of the study were to demonstrate concordance of the EPI-CE test result with the result from tissue biopsy. The study was performed according to the requirements of Good Clinical Practice (ICH E6(R2) and ISO 20916), and clinical data was collected using a standardized Case Report Form (Supplement 1).

### Sample collection

Samples were collected after informed consent at the time of enrollment, stored at + 2 to + 8°C after collection and submitted to the sponsors site (Exosome Diagnostics GmbH, Martinsried, Germany or Exosome Diagnostics, Inc., Waltham, United States), where they were prefiltered and frozen at − 80 °C within 14 days of collection. Informed consent was obtained from all subjects in accordance with the Ethical Principles for Medical Research Involving Human Subjects outlined in the Declaration of Helsinki and the study was approved by a local ethics committee. Urine specimens were accepted if they were first-catch, volume of 10–50 mL, collected at least one hour after last void, without prior digital rectal exam (DRE) and showed no obvious sign of being hemolytic.

### EPI-CE analysis

All samples were sent to Exosome Diagnostic GmbH, Martinsried, Germany, where they were analyzed according to the Instructions for Use (IFU) of the EPI-CE test. The operators executing the testing were blinded to the clinical data of the subjects. The EPI Score was calculated using https://episcore.exosomedx.com from the relative gene expression of the three RNA biomarkers ERG (V-ets erythroblastosis virus E26 oncogene homologs), PCA3 (prostate cancer antigen 3), and SPDEF (SAM Pointed Domain Containing ETS Transcription Factor), without inclusion of any other clinical parameters. The online calculator accepts RT-qPCR data derived using the EPI-CE kit and is accessible to laboratories performing the EPI-CE analysis. The test provides a risk score (scale 0-100), which correlates with the probability of HGPCa (≥GG2) on biopsy^12^.

### Statistics

Sample size was calculated assuming a sensitivity of 92% and specificity of 30% based on previous publications^3,4^ and lower bounds of 74% and 16%, respectively, resulting in a minimum of 35 HGPCa cases (≥ GG2) and 71 controls (i.e., benign or ≤ GG1 cases).

The primary analysis of the study was a cut-point analysis at the previously validated cut point of 15.6 to determine the EPI-CE test performance measures of sensitivity and negative predictive value (NPV). Confidence intervals for these metrics were calculated using the Clopper–Pearson method.

All statistical analyses and plots were generated using Python 3.7 (Python.org, June 2018). Statistical differences in clinical and demographic factors of categorical variables were estimated with a Pearson’s Chi-squared test and continuous variables were subjected to a student’s t-test. DeLong’s test was applied to assess the significance of AUC differences between ROC curves.

### Institutional review board statement

The study was conducted according to the applicable guidelines of the Declaration of Helsinki and approved by local (western) institutional review boards (IRBs; IRB Study Numbers: 1289272 and 1292952; approval dates 03-Aug-2020 and 11-Sep-2020, respectively; IRB Tracking Number: 20202262). Due to the anonymized, non-interventional and non-invasive study design, general Ethics Committee (EC) review and approval was waived for this study upon consultation with the Bavarian EC. Still, the study protocol and Informed Consent Form were submitted to the local EC (Ethics Committee of the LMU Munich; Project Number 17-0509) and approved.

### Informed consent statement

Informed consent was obtained from all subjects involved in the study.

## Results

### Analytical validation and patients characteristics

Prior to the clinical study, the analytical performance of the EPI-CE test was validated according to the requirements for *in-vitro* diagnostics^11^ and documented in the EPI-CE Instructions for Use (IFU) (Supplement 2). Further, a direct split-sample comparison of the performance of the EPI-LDT and the EPI-CE test was performed using 52 urine samples (Supplement 3).

A total of N=124 patients were enrolled from eight clinical sites in Germany (n=56, 45%), two sites in US (n=53, 43%), and one in UK (n=15, 12%). Of these, n=15 (12%) failed to yield an EPI-CE result during processing (eight IVD assay control failures, five exosomal RNA extraction failures, one too low exosomal RNA signal and one operator error), leaving n=109 samples with complete clinical and analytical information. The cohort demographics are shown in Table 1.

**Table 1:** Demographic/clinical characteristics for the total 124 and the final 109 cases in the EPI-CE cohort.

	EPI-CE cohort, total	EPI-CE cohort, final
**Total**	124	109
**Age median, IQR**	66.0 (62.0–72.0)	66.0 (62.0–71.0)
**PSA median, IQR**	5.9 (4.8–7.9)	5.9 (4.8–7.5)
**Family history, n (%)**		
Yes	15 (12.1%)	14 (12.8%)
No	109 (87.9%)	95 (87.2%)
**Ethnicity, n (%)**		
African American	2 (1.6%)	2 (1.8%)
Caucasian	99 (79.8%)	88 (80.7%)
Other	23 (18.6%)	19 (17.4%)
**DRE, n (%)**		
Nonsuspicious	66 (53.2%)	58 (53.2%)
Suspicious	24 (19.4%)	19 (17.4%)
NA	34 (27.4%)	32 (29.4%)
**Grade Group, n (%)**		
Benign	61 (49.2%)	50 (45.9%)
GG 1 (ISUP1, GS3 + 3)	22 (17.7%)	21 (19.3%)
GG 2 (ISUP2, GS3 + 4)	22 (17.7%)	20 (18.4%)
GG 3 (ISUP3, GS4 + 3)	10 (8.1%)	9 (8.3%)
GG 4 (ISUP4, all GS8)	5 (4.0%)	5 (4.6%)
GG 5 (ISUP5, > GS8)	4 (3.2%)	4 (3.7%)
≥ GG 2 (ISUP2, GS3 + 4)	41 (33.1%)	38 (34.9%)

*DRE* digital rectal exam, *GG* Grade Group, *GS* Gleason score, *IQR* interquartile range, *ISUP* International Society of Urological Pathology, *PSA* prostate-specific antigen.

### EPI Score performance and cut-point analysis:

**Figure 1:**
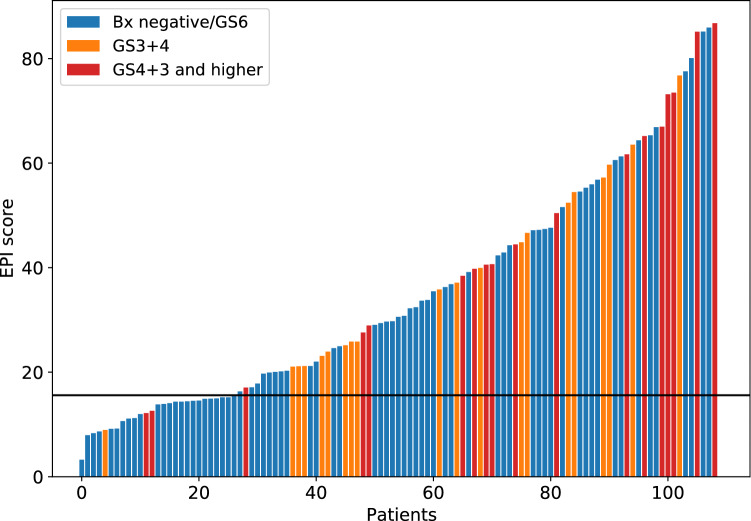
Waterfall plot of all individual subjects in the study, ranked by EPI score and colored by biopsy pathology outcome. The black horizontal line represents the 15.6 cut-point.

The individual EPI scores from all participants in the study is plotted in a waterfall plot in Fig. 1.

With tissue biopsy pathology as the reference test (HGPCa ≥ GG2) and dichotomizing the EPI-CE scores into “EPI positive” and “EPI negative” around the 15.6 cut-point^3^, we found that 25% (95%CI 17–34%) of samples had EPI scores below the 15.6 cut-point and could thus have avoided biopsy based on the EPI-CE test. The EPI-CE test had a sensitivity of 92% (95%CI 79–98%) and an NPV of 89% (95%CI 71–98%) (Table 2).

**Table 2:** Performance characteristics of EPI-CE at the 15.6 cut-point.

	EPI-CE (N = 109), mean (95% CI)
Samples below cut-point	25% (17–34%)
Sensitivity	92% (79–98%)
Specificity	34% (23–46%)
False negative rate (> GG2)	8% (2–21%)
False negative rate (> GG3)	3% (0–14%)
Negative Predictive value	89% (71–98%)
Positive Predictive value	43% (32–54%)

**Figure 2:**
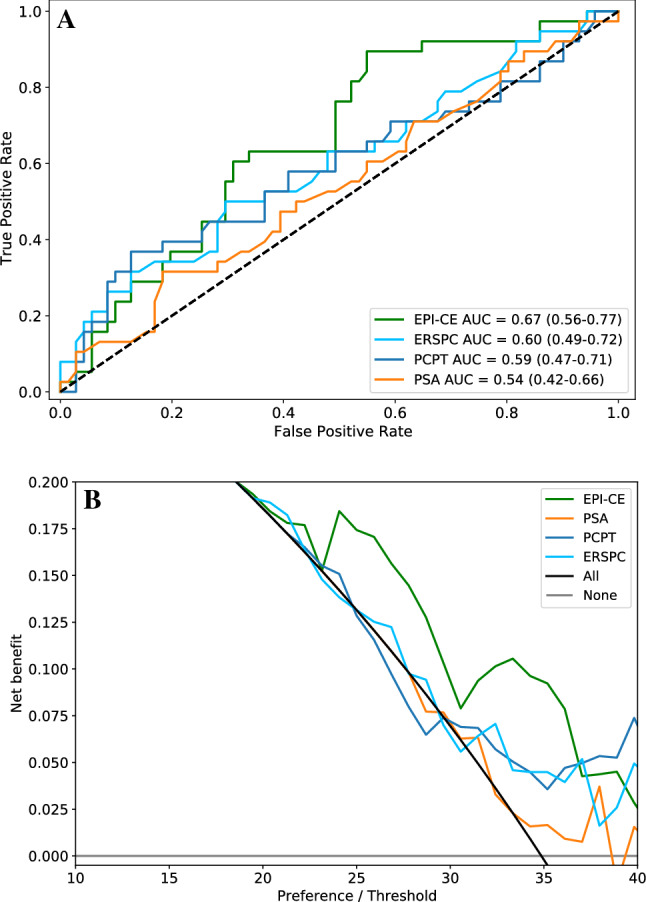
Receiver-Operating-Characteristics Curve and Decision Curve/Net Benefit analysis of EPI-CE compared to PSA and the two multiparametric risk calculators, PCPT and ERSPC, showing that the EPI-CE score provides a higher Area-Under-Curve (AUC) and a higher Net Benefit than either of the multivariate risk calculators across a wide range of risk acceptance levels.

In ROC analysis, the AUC of the EPI-CE Score 0.67 (95% CI 0.56–0.77) was superior to PSA 0.54 (95% CI 0.42–0.66, *p*=0.0926) and both multiparametric risk calculators PCPT 0.59 (95% CI 0.47–0.71, *p*=0.3066) and ERSPC 0.60 (95% CI 0.49–0.72, *p*=0.4079), but failed to reach significance at the current cohort size (Fig. 2A).

In a Decision Curve Analysis^13,14^ (Fig. 2B) we found EPI-CE to provide a higher Net Benefit than any of the alternatives across a wide range of risk acceptance levels compared to PSA and the two multiparametric risk calculators.

## Discussion

The challenges associated with the use of PSA have emphasized the unmet need to develop additional measures for identifying clinically significant PCa while reducing unnecessary biopsies, over-diagnosis of low-risk disease and subsequent over-treatment. These efforts have led to the development of several biomarker assays, most of which incorporate PSA and other existing clinical features, making it difficult to assess the performance of the biomarkers^1,15,16^. This highlights the importance of tests, such as EPI-CE, that provide additional independent information to aid in clinical decision-making^3^.

Until now, the EPI test has only been available in the United States as a central laboratory test. However, different health care systems across Europe and differences in the delivery of clinical diagnostic testing makes a centralized laboratory test impractical in European clinical practice. The study presented here demonstrates that the newly developed EPI-CE IVD test provides similar performance to the previously published EPI-LDT, in a cohort of patients primarily from European clinical sites. This study is the first to report on the performance of the EPI-CE test and the first to demonstrate that exosome-based diagnostic tests can be developed to the highest CE-IVD quality standards and making it accessible to European patients and clinicians.

Although the prevalence of HGPCa, was higher at 35% in the present European cohort vs 30% in previous US cohorts^3,4^, a Chi-squared test on Benign, GG1 and ≥ GG2 cases did not reveal any significant difference (*p*=0.585), with the overall distribution of Grade Groups being very similar (i.e., no particular GG was responsible for the higher prevalence of HGPCa).

The cut-point performance metrics of EPI-CE observed in the present study were also similar to those previously published for EPI-LDT in the US^10^ using the same 15.6 cut-point, with the most important metrics being the sensitivity (92% vs. 92%, to identify as many HGPCa cases as possible), the NPV (89% vs. 90%, providing confidence that patients with a negative test result can defer biopsy) and the number of patients below cut-point (25% vs. 23%, representing a meaningful fraction of patients to benefit by deferred biopsy), for the EPI-CE vs the EPI-LDT, respectively.

The Decision Curve showed that EPI-CE provides a higher Net Benefit than either of the multivariate risk calculators across a wide range of risk acceptance levels, indicating that using the EPI-CE score to determine who to biopsy would lead to an improved clinical outcome.

Similarly, in rank-based analysis, EPI-CE had the highest AUC 0.67 followed by the multivariate risk models ERSPC AUC 0.60 and PCPT AUC 0.59 and PSA with AUC 0.54, although none of these comparisons reached statistical significance. The 95% Confidence Interval of the AUC of EPI-CE was 0.56–0.77 in this study, indicating that at the current cohort size the natural variability of the data set is well within the range of the 0.70 that would be expected based on previous publications of EPI performance^2–5^. Further, as demonstrated in this publication of EPI-CE as well as the previous publications on EPI, the performance is consistently superior to existing alternatives, i.e., the multiparametric risk calculators. It is important to note the improved performance of EPI-CE relative to the multiparametric models that do not include MRI, especially considering the recent PSA screening recommendations from the EAU, which calls for patients to undergo “risk stratification” prior to MRI^1^. We deliberately enrolled patients in the present study who did not undergo MRI, to estimate the performance of EPI in this setting. Analysis of a sub-cohort of patients that meet all the current EAU recommendations for further risk stratification prior to MRI (N = 78, ≥ 3 ng/mL, 50–70 years) revealed no difference in performance relative to the EPI-CE intended use population (2–10 ng/mL, ≥ 50 years), confirming the suitability of the EPI test for risk stratification prior to MRI.

The main limitation to the present study is the limited cohort size, which is partially mitigated by the comparison to previously published performance characteristics of the US EPI-LDT version of the test. Further, since the study was designed to show the performance of the EPI-CE independent of the patient having had an MRI, the absence of MRI data does not allow for direct EPI-CE to MRI comparisons. MRI is an important risk stratification tool for prostate cancer and further studies are needed to show the performance of EPI in various clinical settings relative to MRI.

A major strength of the present study is that it represents a prospective validation, on a clinically relevant European cohort, of the first CE-marked exosome-based urine test for use in clinical laboratories across Europe, adding an important tool in the risk stratification-toolbox for prostate cancer for patients and physicians.

## Conclusions

The EPI-CE test is a non-invasive urine exosomal RNA test for risk stratification of patients under suspicion of prostate cancer. In this analysis, the EPI-CE clinical provides improved performance relative to PSA and the multiparametric risk models (ERSPC and PCPT) for predicting ≥ GG2 cancer. This makes the EPI test ideally suited for “Risk Assessment” prior to MRI as recently recommended by the EAU for Early Detection of Prostate Cancer.

## Supplementary Information

Supplementary Information.
