# Supplementary material for: Validation of a CE-IVD, urine exosomal RNA expression assay for risk assessment of prostate cancer prior to biopsy
Source: Sci Rep. 2022 Mar 21;12:4777. doi: 10.1038/s41598-022-08608-z (PMC8938406; doi:10.1038/s41598-022-08608-z)
Supplement: Supplementary file 1 — Supplementary Information. [file 41598_2022_8608_MOESM1_ESM.pdf]

Validation of a CE-IVD, urine exosome RNA expression assay for risk assessment of prostate cancer prior to biopsy.

Alexander Kretschmer[1], Holger Kajau[2], Eric Margolis[3], Ronald Tutrone[4], Tobias Grimm[5], Matthias Trottmann[6], Christian Stief[1], Georg Stoll[7], Christian A. Fischer[7], Claudia Flinspach[7], Anja Albrecht[7], Lisa Meyer[7], Tina Priewasser[7], Daniel Enderle[7], Romy Müller[7], Phillipp Torkler[7], Jason Alter[7], Johan Skog[7*] and Mikkel Noerholm[7]

1) Ludwig-Maximilians University Munich; alexander.kretschmer@med.uni-muenchen.de
2) SRH Wald-Klinikum Gera GmbH; holger.kujau@srh.de
3) Department of Urology Hackensack Meridian School of Medicine; emargolis@njurology.com
4) Chesapeake Urology Associates; rtutrone@uniteduro.com
5) Urologische Gemeinschaftspraxis Kaufbeuren; grimm@urologie-kaufbeuren.de
6) Urologie und Andrologie am Promenadeplatz
7) Exosome Diagnostics, a Bio-techne brand, Waltham, MA, USA

*Correspondence: J.S., johan.skog@bio-techne.com

SUPPLEMENT INFORMATION:

Supplement 1

Case Report Form – Clinical Evaluation of ExoDx™ Prostate(IntelliScore) in Men Presenting for Initial Prostate Biopsy

Supplement 2

Analytical Validation of EPI-CE

Supplement 3

Technical comparison EPI-CE vs EPI-LDT

Subject ID Number, or barcode sticker

CASE REPORT FORM

PART 1: STUDY SITE VISIT DATE

Date of patient enrolment visit: D D M M M Y Y Y Y

INFORMED CONSENT

Has the subject:

Read and understood the patient information leaflet?	☐ YES	☐ NO
Completed an informed consent sheet for the study?	☐ YES	☐ NO
Retained copies of the leaflet and completed consent sheet?	☐ YES	☐ NO

INCLUSION CRITERIA

Is/does the subject:

A male aged 50 years or above?	☐ YES	☐ NO
Scheduled for a prostate biopsy?	☐ YES	☐ NO
Have a PSA between 2-10 ng/mL?	☐ YES	☐ NO

EXCLUSION CRITERIA

Has/does the subject:

Been diagnosed with hepatitis (any type) and/or HIV?	☐ YES	☐ NO
Have a history of renal/bladder tumours in the last 6 months?	☐ YES	☐ NO
Received neoadjuvant hormonal (ADT) or radiation therapy the last 6 months?	☐ YES	☐ NO
Clinical symptoms of urinary tract infection (including prostatitis) at the time of enrolment?	☐ YES	☐ NO
Had a prostate MRI prior to biopsy?	☐ YES	☐ NO

Completed by: ______________ ______________ ______________

Name *Signature* *Date (DD/MMM/YYYY)*

FIRST CATCH URINE COLLECTION

Date and time of urine collection: D D M M M Y Y Y Y

H H M M

Time of previous urination/void of the day H H M M — N/A (if first void of day)

Is the specimen **first catch** urine? YES NO
If NO, withdraw subject from study

Is the specimen **volume between 10-50 mL**? YES NO
If NO, withdraw subject from study

Was the sample **donated directly into** the specimen container provided by/agreed upon with the sponsor? YES NO
If NO, withdraw subject from study

Is the specimen **catheterized or grossly bloody**? YES NO
If YES, withdraw subject from study

Was Digital Rectal Examination **(DRE) performed** since last void? YES NO
If YES, withdraw subject from study

Is urine **collection within 4 weeks prior** to the scheduled biopsy? YES NO
If NO, withdraw subject from study

*NOTE **COLLECTION DATE AND SUBJECT ID** (OR BARCODE STICKER) ON THE SPECIMEN CONTAINER*
*STORE SAMPLES AT **2-8 °C** UNTIL COLLECTION. DO **NOT** STORE SAMPLES FOR LONGER THAN **10 DAYS***

COMPLETE PART 1 OF CRF AND KEEP TOGETHER WITH URINE SAMPLE
CONTACT EXOSOME DIAGNOSTICS TO ARRANGE PICKUP/SHIPPING OF SAMPLE + CRF PART 1

Completed by: ____________________ ____________________ ____________________
Name *Signature* *Date (DD/MMM/YYYY)*

PATIENT CLINICAL HISTORY

Year of birth:	Y Y Y Y
Date of last PSA test:	D D M M M Y Y Y Y
PSA level at date of last PSA test:	. ng/mL
Was the result of Digital Rectal Examination (DRE) suspicious?	YES / NO / N/A
Is there a family history of prostate cancer?	YES / NO<br>*If YES, indicate all that apply below*<br>Brother / Father / Grandfather<br>Uncle / Other
Has the subject received invasive treatments for benign prostatic hypertrophy (BPH) or lower urinary tract symptoms within 6 months prior to study enrollment?	YES / NO
Patient ethnicity	(type one letter)<br>(C) Caucasian, (B) Black/African, (A) Asian, (O) Other/Unknown/Not stated

PRINICIPAL INVESTIGATOR DECLARATION – PART 1

I AM CONFIDENT THAT THE INFORMATION SUPPLIED IN THIS CASE REPORT FORM IS COMPLETE AND ACCURATE.
I CONFIRM THAT WRITTEN INFORMED CONSENT WAS OBTAINED PRIOR TO THE STUDY.

Name:		Practice/Clinic:	
Signature:		Date:	D D M M M Y Y Y Y

RETURN COMPLETED CRF PART 1 TOGETHER WITH SAMPLE TO EXOSOME DIAGNOSTICS

SAMPLE ACCESSIONING NOTES – FOR EXOSOME DIAGNOSTICS USE ONLY		
**Received By:**	**Date/Time Received:**	**Color:**
**# Filters used:**	**Total Sample Volume:**	**Clarity:**
**ExoDx Barcode 1:**	**ExoDx Barcode 2:**	**ExoDx Barcode 3:**
**Aliquot Volume 1:**	**Aliquot Volume 2:**	**Aliquot Volume 3:**
**Data double checked by:**<br>(signature of 2nd Operator after double check and scan of the CRF)		

PART 2: COMPLETE WHEN BIOPSY OUTCOME IS AVAILABLE

Date of biopsy: D D M M M Y Y Y Y

☐ N/A

If N/A, withdraw subject from study

What type of biopsy was performed? ☐ TRUS ☐ Fusion ☐ Other

If OTHER, indicate type below

Was the biopsy an initial or repeat biopsy? ☐ Initial ☐ Repeat

If REPEAT, indicate no. of previous biopsies below

☐ #previous biopsies (NOT including this one)

Did the subject take antibiotics in the **4 weeks prior** to biopsy? ☐ YES ☐ NO

If YES, indicate type(s) below

(1)	(2)

Did the subject take medication known to affect serum PSA levels in the **6 months prior** to biopsy (e.g. Finasterid)? ☐ YES ☐ NO

If YES, indicate type(s) below

(1)	(2)
(3)	(4)

De-identified Histology Report attached? ☐ YES ☐ NO

If NO, provide details in the section below

Completed by: ______________________ ______________________ ______________________
Name *Signature* *Date (DD/MMM/YYYY)*

HISTOLOGY INFORMATION (ONLY IF DE-IDENTIFIED HISTOLOGY REPORT IS NOT ATTACHED)

How many cores were removed during the biopsy? ☐☐ cores

Was high-grade prostatic intraepithelial neoplasia (HGPIN) observed? ☐ YES ☐ NO

If YES, indicate no. of cores below

☐☐ cores

Was atypical small acinar proliferation (ASAP) observed? ☐ YES ☐ NO

If YES, indicate no. of cores below

☐☐ cores

Was prostate cancer confirmed? ☐ YES ☐ NO

If YES, complete the information below

Number of prostate cancer positive cores: ☐☐ cores

Gleason grade: ☐ Primary *(3-5)* ☐ Secondary *(3-5)* ☐ Total *(6-10)*

PRINCIPAL INVESTIGATOR DECLARATION – PART 2

I AM CONFIDENT THAT THE INFORMATION SUPPLIED IN THIS CASE REPORT FORM IS COMPLETE AND ACCURATE.
I CONFIRM THAT WRITTEN INFORMED CONSENT WAS OBTAINED PRIOR TO THE STUDY.

Name:		Practice/Clinic:	
Signature:		Date:	*D D M M M Y Y Y Y*

RETURN THE COMPLETED CRF PART 2 TOGETHER WITH THE HISTOLOGY REPORT TO EXOSOME DIAGNOSTICS PER MAIL OR E-MAIL

DATA TRANSFER NOTES – FOR EXOSOME DIAGNOSTICS USE ONLY	
**Data double checked by:**<br>(signature of 2nd Operator after double check and scan of the CRF)	

Supplement 2

Analytical Sensitivity

- ### Limit of Blank (LOB); Limit of Detection (LOD)

The LOB and LOD for each EPI-CE detection assay was determined in line with the principles outlined in CLSI EP17-A2[5], utilizing dilution series of the target analytes from 100 down to 0 copies of in-vitro transcribed RNA with a total 60 replicates per analyte concentration. The data was collected over 5 days using 3 independent lots of EPI-CE Assay Kit reagents.
The analyte level yielding hit rates of ≥95% (LOD) for all EPI-CE assays was determined at 6.25 copies of RNA per RT-qPCR reaction. None of the analyte-naïve samples yielded any measurable signals (LOB). The combined results are depicted in Table 1.

RNA (copies/reaction)	Positive vs. Total Results				Hit Rate			
	ERG	PCA3	SPDEF	IPC	ERG	PCA3	SPDEF	IPC
**0**	0/15	0/15	0/15	0/45	0%	0%	0%	0%
**1.56**	38/60	36/60	36/60	115/180	63%	60%	60%	64%
**6.25**	57/60	60/60	60/60	179/180	95%	100%	100%	100%
**12.5**	60/60	60/60	60/60	180/180	100%	100%	100%	100%
**25**	60/60	60/60	60/60	180/180	100%	100%	100%	100%
**50**	60/60	60/60	60/60	180/180	100%	100%	100%	100%
**100**	60/60	60/60	60/60	180/180	100%	100%	100%	100%

Table 1 Limit of Blank and Limit of Detection determined for the EPI-CE RT-qPCR assays on a serial dilution of in-vitro transcribed target RNAs.

- ### Limit of Quantitation (LOQ)

The LOQ for each EPI-CE detection assay was determined in line with the principles outlined in CLSI EP17-A2[5], utilizing dilution series of the target analytes from 100 down to 12.5 copies of in-vitro transcribed RNA with a total 60 replicates per analyte concentration. The data was collected over 5 days using 3 independent lots of EPI-CE Assay Kit reagents.
The analyte level yielding hit rates of 100% and not exceeding the predefined precision goal (SD = 0.75 CTs; LOQ) was determined at 12.5 copies of RNA per RT-qPCR reaction for the ERG, PCA3 and IPC assay, while the SPDEF met the acceptance criterion with analyte concentration of ≥25 copies of RNA per RT-qPCR reaction. The combined results are depicted in Table 2.

RNA (copies/reaction)	Standard Deviation (SD; 60 replicates)				Hit Rate			
	ERG	PCA3	SPDEF	IPC	ERG	PCA3	SPDEF	IPC
**12.5**	0.69	0.69	0.84	0.39	100%	100%	100%	100%
**25**	0.52	0.41	0.52	0.28	100%	100%	100%	100%
**50**	0.36	0.43	0.39	0.24	100%	100%	100%	100%
**100**	0.28	0.30	0.25	0.14	100%	100%	100%	100%

Table 2 Limit of Quantitation determined for the EPI-CE RT-qPCR assays on a serial dilution of in-vitro transcribed target RNAs.

- ### Linear Range

The Linear Range for each EPI-CE detection assay was determined in line with the principles outlined in CLSI EP06-A[2], utilizing dilution series of the target analytes from 10^6 down to LOQ-corresponding copies of in-vitro transcribed RNA, spanning 7 (SPDEF) and 8 (ERG, PCA3, IPC) dilution points. The data was collected over 5 days using 3 independent lots of EPI-CE Assay Kit reagents. 60 replicates for the concentrations ≤ 100 copies of RNA and 15 replicates for concentrations > 100 copies of RNA were utilized to determine each assay's linear range by regression analysis.

All EPI-CE assays showed linearity indicated by R^2 values of ≥ 0.985 from LOQ up to 10^6 copies of analyte RNA. The combined results are depicted in Table 3 and Figure 1.

Assay	Tested Range [RNA copies / Reaction]	R^2
**ERG**	12.5 – 10^6	0.9901
**PCA3**	12.5 – 10^6	0.9901
**SPDEF**	25 – 10^6	0.9932
**IPC**	12.5 – 10^6	0.9966

Table 3 Linear Ranges determined for the EPI-CE RT-qPCR assays on a serial dilution of in-vitro transcribed target RNAs.

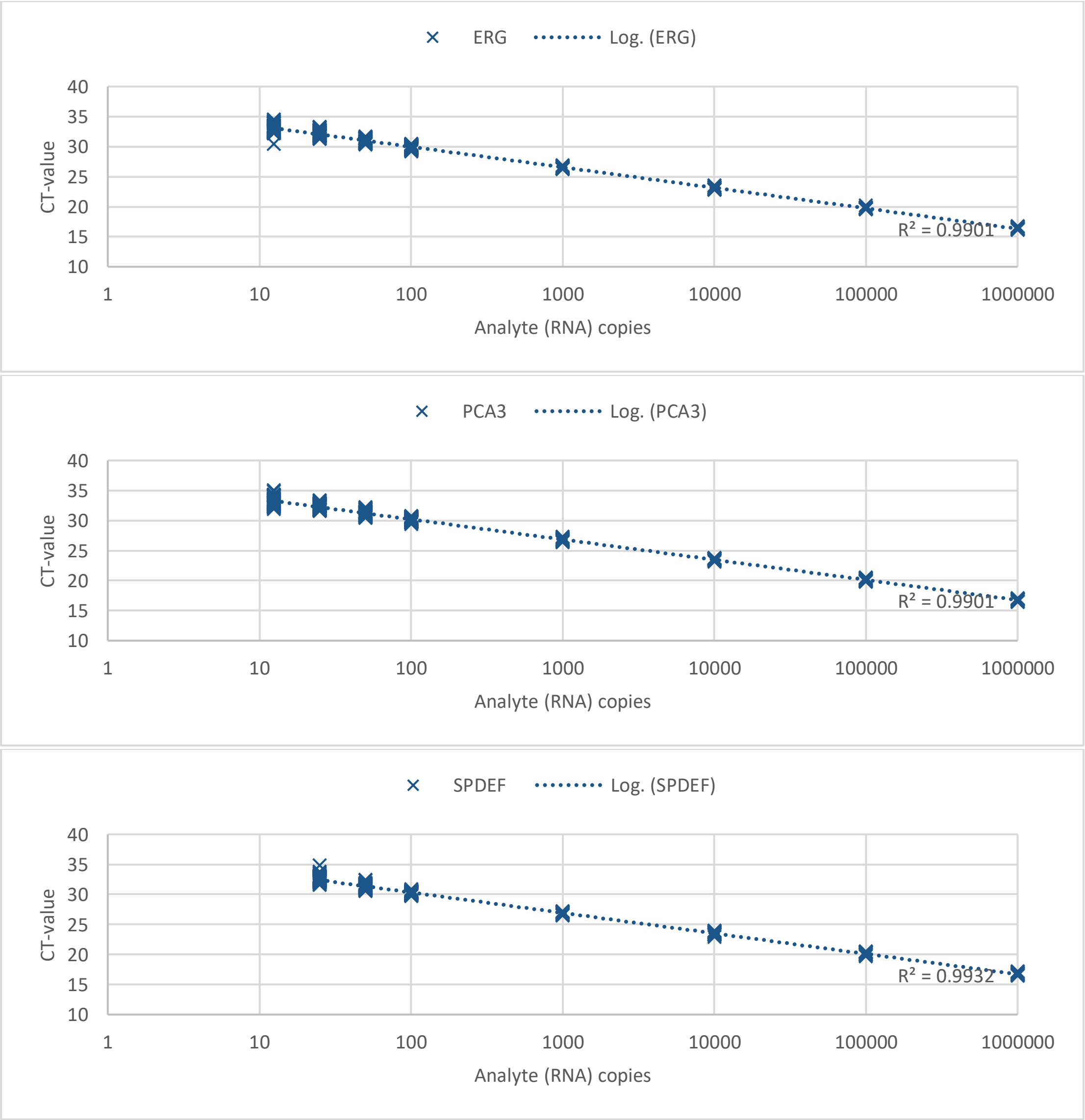

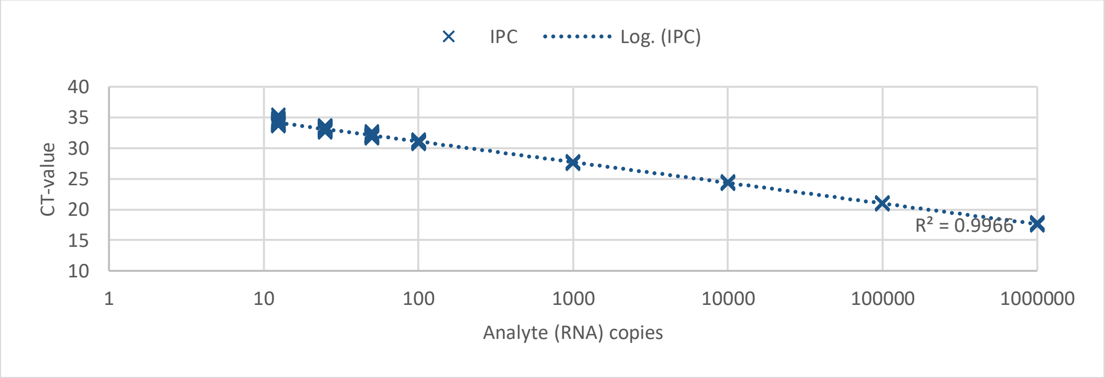

Figure 1 Linear Ranges determined for the EPI-CE RT-qPCR assays on a serial dilution of in-vitro transcribed target RNAs.

Analytical Specificity

- ### Interfering Substances

The agents listed in Table 4 were added in the indicated final concentrations to aliquots of unprocessed, pooled male urine. 6 replicates of test-agent supplemented samples along with 6 replicates of non-supplemented samples were subjected to the EPI-CE procedure (including pre-processing) for paired difference testing (PDT) in line with the principles outlined in CLSI EP07, 3rd ed[3]. Whenever addition of an agent required a solvent, 6 replicates of solvent control were included in the PDT setup. In addition, agents potentially introduced during the EPI-CE process were tested using the identical approach, by direct addition to the RNA eluates or RT-qPCR reaction. Table 5 lists any agent that qualified as interferents in the PDT along with their critical threshold levels determined by a dose response titration, where 4 dilutions of the interferent were evaluated in addition to the PDT concentrations.

Category	Substance	Test Concentration
**Urine Constituents**	Albumin	400 mg/L
	Bilirubin	60 mg/L
	Calcium	800 mg/L
	Cholesterol	700 mg/L
	Glucose	400 mg/L
	Hemoglobin	N/A (tested with whole blood)
	IgG	20 mg/L
	Sodium Chloride	8 g/L
	Triglycerides	15 g/L
	Uric Acid	1.2 g/L
	Microorganisms	104 CFU/mL
	White blood cells	N/A (tested with whole blood)
	Whole blood	0.05%
**Pharmacologically Active Agents**	Alcohol (Ethanol)	4.65 mg/mL
	Caffeine	101.4 µM
**Processing-Related Agents**	Chaotropic Salt (GTC*)	1 mM (in RT-qPCR)
	Alcohol (Ethanol)	20% (in RT-qPCR)
	DNase	0.05 µg (50U/mg) in RNA eluate
	RNase	0.05 µg (50U/mg) in RNA eluate

Table 4 Substances and their concentrations tested for interference with the EPI-CE Assay by paired-difference testing. * = Guanidine thiocyanate

Category	Interferent	Critical Concentration	IPC Failure
**Urine Constituents**	Bilirubin	> 6 mg/L	YES
	Calcium	> 80 mg/L	YES
	Triglycerides	> 1.5 g/L	NO*
	Alcohol (Ethanol)	> 2.5 % (v/v; in RT-qPCR)	YES

Processing-Related Agents	RNase	> 0.005 ng (in RNA Eluate)	YES

Table 5 Substances tested positive for interference with the EPI-CE Assay in the paired difference testing and their critical threshold levels determined by dose-response titrations. The "IPC Failure" column indicates if the interference is identified by the IPC. * = High Triglyceride levels are not a specific inhibitor of the EPI-CE process but generally incompatible with exosome isolations from urine, compromising the sample's exosome content. Consequently, physiological conditions leading to elevated Triglyceride levels in urine samples (Chyluria[4]) must be incompatible with the EPI-CE procedure (see also the "Limitations" section).

- Cross-Reactivity

Cross-reactivity of the EPI-CE Assay was assessed in silico on the human genome and the genomes from the most common microorganisms associated with urinary tract infections, E. coli (bacteria)[6] and C. albicans (fungi)[7]. In addition, cross-reactivity with human genomic DNA (up to 5000 copies per reaction) and non-target RNAs was functionally assessed. None of the investigations resulted in any significant cross-reactivities.

Analytical Precision

- Repeatability

Repeatability of each EPI-CE RT-qPCR assay was determined in line with the principles outlined in CLSI EP05-A3[1] using a 3-member panel of representative male urine samples with low, medium and high EPI-CE target RNA analyte levels. RNA from each urine sample was isolated in 2 runs per day with 2 replicates per run over 20 processing days on a single site by a single operator on a single qPCR instrument with a single lot of EPI-CE reagents. Table 6 depicts the resulting, nested ANOVA derived repeatability and within-laboratory precision for each EPI-CE RT-qPCR assay by means of the corresponding standard deviation (SD) and coefficient of Variability in % (%CV) for each sample and assay combination. The corresponding EPI-CE Score results are depicted in the analogous in Table 7.

EPI-CE Assay	Sample (RNA Expression Level)	Mean [CT]	Repeatability		Within-Laboratory Precision	
			SD	%CV	SD	%CV
ERG	Low	34.4	0.9	2.6	1.0	3.0
	Medium	33.4	0.6	1.9	1.0	3.0
	High	30.6	0.3	0.9	0.5	1.5
PCA3	Low	29.7	0.2	0.6	0.6	1.9
	Medium	28.5	0.1	0.4	0.3	1.2
	High	27.2	0.1	0.3	0.4	1.4
SPDEF	Low	25.9	0.1	0.3	0.3	1.3
	Medium	25.1	0.1	0.3	0.5	1.9
	High	24.3	0	0.2	0.2	1.0
IPC	Low	29.4	0.1	0.2	0.6	2.2
	Medium	29.5	0.1	0.2	0.5	1.8
	High	29.2	0.1	0.2	0.4	1.4

Table 6 Repeatability and within Within-Laboratory precision of the EPI-CE procedure assessed for each RT-qPCR assay on 3 representative urine samples with high, medium and low biomarker expression levels.

Sample (RNA Expression Level)	Mean [Score]	Repeatability		Within-Laboratory Precision	
		SD	%CV	SD	%CV
**Low**	49.4	7.3	14.7	7.5	15.2
**Medium**	34.9	4.7	13.4	4.9	13.9
**High**	64.2	2.4	3.7	3.2	4.9

Table 7 Repeatability and within Within-Laboratory precision of the EPI-CE procedure assessed for on 3 representative urine samples with high, medium and low biomarker expression levels.

- Reproducibility

Reproducibility of each EPI-CE RT-qPCR assay was determined using a 3-member panel of representative male urine samples with low, medium and high EPI-CE target RNA analyte levels. RNA from each urine sample was isolated in a single run per day with 2 replicates per run over 5 processing days on 2 sites by 4 operators (2 per site) on 4 qPCR instruments (2 per site) with a single lot of EPI-CE reagents. Table 8 depicts the resulting, nested ANOVA derived repeatability and precision values for each EPI-CE RT-qPCR assay by means of their corresponding standard deviation (SD) and coefficient of variability in % (%CV) for each sample and assay combination. The corresponding EPI-CE Score results are depicted in the analogous in Table 9.

EPI-CE Assay	Sample (RNA Expression Level)	Mean [CT]	Intra-Day		Inter-Day		Inter-Instrument		Inter-Operator		Inter-Site		Total	
			SD	%CV	SD	%CV	SD	%CV	SD	%	SD	%CV	SD	%CV
ERG	Low	34.3	1.0	2.8	0.5	1.4	0.3	0.9	0.4	1.0	0.3	0.9	1.2	3.6
	Medium	33.2	0.5	1.4	0.7	1.9	0.2	0.5	0.4	1.3	0.1	0.2	0.9	2.8
	High	30.3	0.2	0.5	0.2	0.5	0.2	0.5	0.2	0.6	0.5	1.4	0.6	2.0
PCA3	Low	29.6	0.2	0.6	0.2	0.6	0.3	0.8	0.2	0.6	0	0	0.5	1.5
	Medium	28.4	0.1	0.4	0.3	1.0	0.2	0.6	0.2	0.7	0.0	0.0	0.5	1.7
	High	27.0	0.1	0.2	0.3	0.8	0.3	0.7	0.1	0.4	0.3	0.9	0.5	1.8
SPDEF	Low	25.9	0.1	0.2	0.3	0.9	0.0	0.0	0.3	0.8	0	0	0.4	1.7
	Medium	25.0	0.1	0.2	0.2	0.6	0.1	0.2	0.3	1.0	0	0	0.4	1.7
	High	24.1	0	0.1	0.2	0.5	0.2	0.4	0.2	0.5	0.2	0.5	0.3	1.4
IPC	Low	29.2	0.1	0.2	0.4	1.2	0	0	0.2	0.7	0	0	0.5	1.7
	Medium	29.2	0.1	0.2	0.2	0.7	0.2	0.6	0.1	0.4	0.1	0.2	0.4	1.3
	High	28.9	0.1	0.2	0.3	0.9	0.2	0.5	0.2	0.5	0.3	0.8	0.5	1.7

Table 8 Reproducibility of the EPI-CE procedure assessed for each RT-qPCR assay on 3 representative urine samples with high, medium and low biomarker expression levels.

Sample (RNA Expression Level)	Mean [Score]	Intra-Day		Inter-Day		Inter-Instrument		Inter-Operator		Inter-Site		Total	
		SD	%CV	SD	%CV	SD	%CV	SD	%	SD	%CV	SD	%CV
Low	50.4	7.6	15.1	3.6	7.0	3.2	6.3	0	0	5.9	11.6	10.7	21.3
Medium	35.4	3.5	9.8	3.8	10.9	2.7	7.6	0	0	3.2	9.0	6.7	18.8
High	65.0	1.3	1.9	2.4	3.6	0.2	0.4	0	0	2.9	4.4	4.0	6.1

Table 9 Reproducibility of the EPI-CE procedure assessed on 3 representative urine samples with high, medium and low biomarker expression levels.

Supplement 3

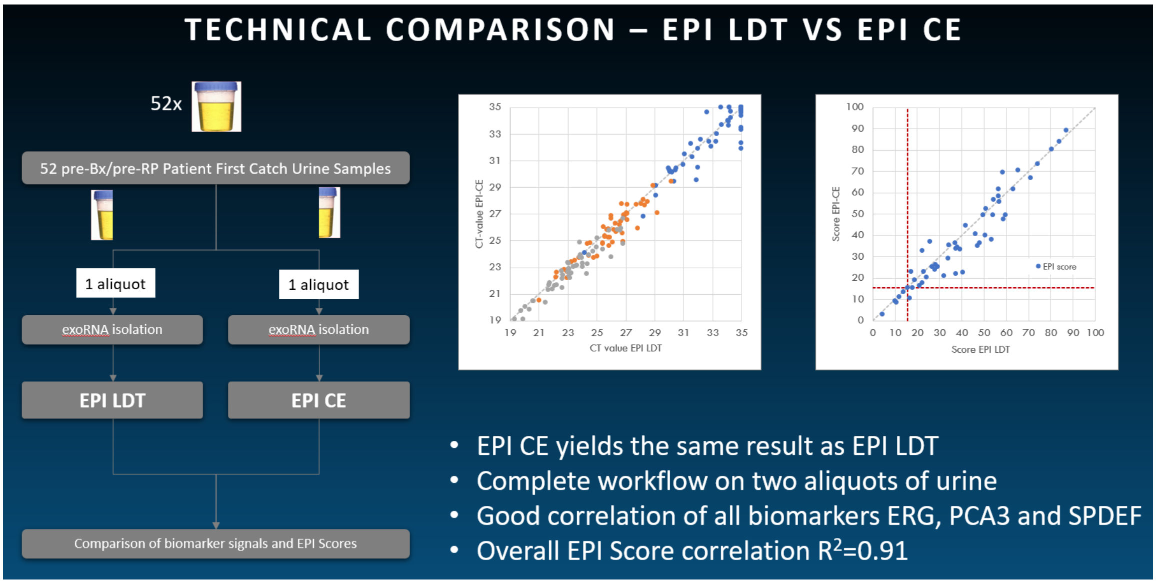

Supplement 3: Direct comparison of EPI-CE and EPI LDT using 52 urine samples with two aliquots processed through the two workflows.

Supplement References

1. CLSI. Evaluation of Precision of Quantitative Measurement Procedures; Approved Guideline—Third Edition. CLSI document EP05-A3. Wayne, PA: Clinical and Laboratory Standards Institute; 2014.
2. NCCLS. Evaluation of the Linearity of Quantitative Measurement Procedures: A Statistical Approach; Approved Guideline. NCCLS document EP06-A. NCCLS, 940 West Valley Road, Suite 1400, Wayne, Pennsylvania 19087-1898 USA, 2003.
3. CLSI. Interference Testing in Clinical Chemistry. 3rd ed. CLSI guideline EP07. Wayne, PA: Clinical and Laboratory Standards Institute; 2018.
4. Abeygunasekera AM, Sutharshan K, Balagobi B. New developments in chyluria after global programs to eliminate lymphatic filariasis. *Int J Urol*. 2017;24(8):582-588. doi:10.1111/iju.13378
5. CLSI. Evaluation of Detection Capability for Clinical Laboratory Measurement Procedures; Approved Guideline—Second Edition. CLSI document EP17-A2. Wayne, PA: Clinical and Laboratory Standards Institute; 2012.
6. Flores-Mireles, Ana L et al. “Urinary tract infections: epidemiology, mechanisms of infection and treatment options.” *Nature reviews. Microbiology* vol. 13,5 (2015): 269-84. doi:10.1038/nrmicro3432
7. Behzadi, Payam et al. “Urinary Tract Infections Associated with *Candida albicans*.” *Maedica* vol. 5,4 (2010): 277-9.
